# Germline and Somatic Whole-Exome Sequencing Identifies New Candidate Genes Involved in Familial Predisposition to Serrated Polyposis Syndrome

**DOI:** 10.3390/cancers13040929

**Published:** 2021-02-23

**Authors:** Yasmin Soares de Lima, Coral Arnau-Collell, Marcos Díaz-Gay, Laia Bonjoch, Sebastià Franch-Expósito, Jenifer Muñoz, Leticia Moreira, Teresa Ocaña, Miriam Cuatrecasas, Cristina Herrera-Pariente, Sabela Carballal, Lorena Moreno, Aránzazu Díaz de Bustamante, Antoni Castells, Luis Bujanda, Joaquín Cubiella, Daniel Rodríguez-Alcalde, Francesc Balaguer, Sergi Castellví-Bel

**Affiliations:** 1 Centro de Investigación Biomédica en Red de Enfermedades Hepáticas y Digestivas (CIBERehd), Gastroenterology Department, Institut d’Investigacions Biomèdiques August Pi i Sunyer (IDIBAPS), Hospital Clínic, 08036 Barcelona, Spain; soaresdeli@clinic.cat (Y.S.d.L.); arnau@clinic.cat (C.A.-C.); bonjoch@clinic.cat (L.B.); franches@mskcc.org (S.F.-E.); jenifer.munoz@ciberehd.org (J.M.); lmoreira@clinic.cat (L.M.); mocana@clinic.cat (T.O.); cristina.herrera@ciberehd.org (C.H.-P.); carballal@clinic.cat (S.C.); lomoreno@clinic.cat (L.M.); castells@clinic.cat (A.C.); fprunes@clinic.cat (F.B.); 2 Moores Cancer Center, Department of Cellular and Molecular Medicine, Department of Bioengineering, University of California, San Diego, La Jolla, CA 92093, USA; mdiazgay@health.ucsd.edu; 3 Centro de Investigación Biomédica en Red de Enfermedades Hepáticas y Digestivas (CIBERehd), Pathology Department, Institut d’Investigacions Biomèdiques August Pi i Sunyer (IDIBAPS), Tumor Bank-Biobank, Hospital Clínic, 08036 Barcelona, Spain; MCUATREC@clinic.cat; 4 Genetics Unit, Hospital Universitario de Móstoles, 28935 Madrid, Spain; arandiazbus@yahoo.es; 5 Gastroenterology Department, Hospital Donostia-Instituto Biodonostia, Centro de Investigación Biomédica en Red de Enfermedades Hepáticas y Digestivas (CIBERehd), Basque Country University (UPV/EHU), 20014 San Sebastián, Spain; LUIS.BUJANDAFERNANDEZDEPIEROLA@osakidetza.eus; 6 Gastroenterology Department, Complexo Hospitalario Universitario de Ourense, Instituto de Investigación Sanitaria Galicia Sur, Centro de Investigación Biomédica en Red de Enfermedades Hepáticas y Digestivas (CIBERehd), 32005 Ourense, Spain; Joaquin.Cubiella.Fernandez@sergas.es; 7 Digestive Disease Section, Hospital Universitario de Móstoles, 28935 Móstoles, Spain; drodrigueza@salud.madrid.org

**Keywords:** serrated polyposis syndrome, genetic predisposition to disease, colorectal cancer, whole-exome sequencing, mutational signatures

## Abstract

**Simple Summary:**

Cancer is the second leading cause of death worldwide. Serrated polyposis syndrome (SPS) is characterized by the presence of serrated lesions in the colon and a higher colorectal cancer (CRC) risk. An important part of risk is due to the alteration of certain genes, which will be transmitted from one generation to another in the same family. Our main objective was to identify alterations of the human genome relevant to the hereditary predisposition to SPS, by focusing on families with several cases of this disease (familial SPS) and by using massive sequencing techniques to decode the genome. Our strategy allowed us to suggest the implication of 14 new genes in SPS predisposition. Identifying the inherited genetic factors involved in SPS can be useful to identify those families with medium-high CRC risk and, therefore, implement more targeted, intensive preventive measures for this group of patients.

**Abstract:**

The serrated polyposis syndrome (SPS) is the most common and yet underdiagnosed colorectal polyposis syndrome. It is characterized by multiple and/or large colonic serrated polyps and a higher associated risk for colorectal cancer (CRC). The main objective of this study was to identify new candidate genes involved in the germline predisposition to SPS/CRC. Thirty-nine SPS patients from 16 families (≥2 patients per family) were recruited without alterations in well-known hereditary CRC genes, and germline and somatic whole-exome sequencing were performed. Germline rare variants with plausible pathogenicity, located in genes involved in cancer development, senescence and epigenetic regulation were selected. Somatic mutational profiling and signature analysis was pursued in one sample per family, when possible. After data filtering, *ANXA10*, *ASXL1*, *CFTR*, *DOT1L*, *HIC1*, *INO80*, *KLF3*, *MCM3AP*, *MCM8*, *PDLIM2*, *POLD1*, *TP53BP1*, *WNK2* and *WRN* were highlighted as the more promising candidate genes for SPS germline predisposition with potentially pathogenic variants shared within families. Somatic analysis characterized mutational profiles in advanced serrated polyps/tumors, revealing a high proportion of hypermutated samples, with a prevalence of clock-like mutational signatures in most samples and the presence of DNA mismatch repair-defective signatures in some cases. In conclusion, we identified new candidate genes to be involved in familial SPS. Further functional studies and replication in additional cohorts are required to confirm the selected candidates.

## 1. Introduction

Colorectal cancer (CRC) is one of the most common cancers worldwide with a significant associated mortality. Aside from lung cancer, with an avoidable environmental cause, CRC is responsible for more deaths than any other malignancy in Western countries [1]. In Europe, CRC represents the second leading cancer type both in incidence and mortality, when considering both genders [2].

A vast majority of CRC cases develop from non-malignant precursor adenomas [3]. In recent years, serrated polyps have been identified as another type of precancerous lesions. They were considered indolent until the discovery of the serrated pathway, which is currently known to be involved in up to 30% of CRC cases [4].

Serrated polyposis syndrome (SPS) is a recent clinical condition characterized by the colonic presence of multiple and/or large serrated polyps, as well as higher CRC risk. In SPS, polyps show characteristics that make them different from sporadic serrated polyps, such as a higher proportion of alterations in *KRAS* and *BRAF*. There is no gender predominance for SPS and the age at diagnosis is 50–60 years [5]. Although its real prevalence is unknown, it could be higher than expected according to data from CRC screening programs [6]. It is also probably under-recognized in the medical community due to the more difficult endoscopic detection of serrated polyps (small size and flat morphology). Therefore, the following criteria were initially established by the World Health Organization (WHO) [7] to help to identify this clinical entity:

(1) at least 5 serrated polyps proximal to the sigmoid colon with two or more of these being >10 mm,
(2) any number of serrated polyps proximal to the sigmoid colon in an individual who has a first-degree relative with serrated polyposis,
(3) >20 serrated polyps of any size, but distributed throughout the colon.

This initial arbitrary definition, recently updated to include also distal serrated polyps and not include the second criterion [8], is not based in any genetic alteration and has been considered by some means restrictive, leading to the under-diagnosis of SPS. Besides, this syndrome surely represents a disease spectrum with a high degree of heterogeneity.

As for other complex diseases, SPS etiology involves genetic and environmental factors. In contrast to conventional adenomas, older age and male sex are not strong risk factors. Risk does not appear to increase substantially in patients over 50 years old, and men and women have roughly equivalent risk. Among modifiable risk factors, as is the case for CRC, smoking, alcohol consumption and obesity seems to be the more relevant factors linked to a higher SPS risk [9].

In some cases, SPS presents a certain degree of familial aggregation, suggesting the existence of germline predisposition to this disease. However, the hereditary basis of this disease is mostly unknown. Some of the common, low-penetrance CRC genetic variants identified through genome-wide association studies could be involved in some SPS cases [10]. Concerning rare, high-penetrance germline predisposition, only *RNF43* has been proposed as a hereditary SPS gene with some controversy and most likely corresponds to a rare contributor [11,12,13]. Other genes such as *BMPR1A, SMAD4, PTEN, MUTYH* or *GREM1* involved in the gastrointestinal hamartomatous polyposis, hereditary mixed polyposis and *MUTYH*-associated polyposis syndromes are not commonly altered in individuals with SPS [14].

Whole-exome sequencing (WES) has been one of the most common strategies to discover new genes associated to hereditary diseases in the last decade [15]. In CRC research, it has been applied both to somatic and germline samples [16,17] permitting a deeper understanding of this disease. Paired germline and somatic WES data can be used to identify new candidate predisposition genes affected by a two-hit inactivation [18]. Also, somatic WES has enormous potential to expand our comprehension of the alterations occurring in tumors or precursor lesions, and it has been used to characterize the mutational burden and somatic mutational spectrum in polyposis and CRC [19].

Accordingly, with the aim of finding rare, high-penetrance germline variants involved in SPS predisposition, we performed germline and somatic WES in 16 families showing familial aggregation mainly compatible with an autosomal dominant pattern of inheritance.

## 2. Results

### 2.1. Patients

**Figure 1:**
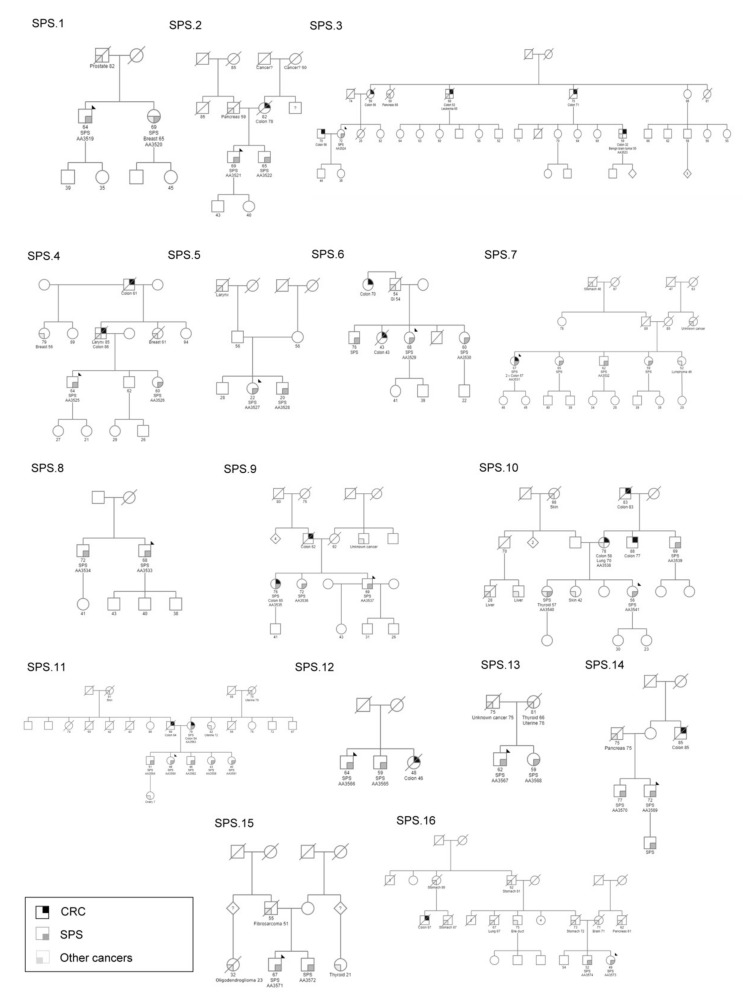
Family trees of the 16 serrated polyposis syndrome families indicated in the study. A black arrow indicates the index case in each family. Current age is specified (for age of onset see Table 1). SPS, serrated polyposis syndrome; CRC, colorectal cancer. Bile duct, brain, breast, colon, GI (gastrointestinal), larynx, liver, ovary, pancreas, prostate, skin, stomach, thyroid and uterine refer to the type of cancer.

Sixteen families including 39 patients (≥2 patients per family) diagnosed with SPS fulfilling the 2010 WHO recruitment criteria were included in the study (Figure 1). Most families (13/16) included two patients per family, whereas SPS.9, SPS.10 and SPS.11 included three, four and six patients, respectively. A complete clinical characterization of the studied cohort is summarized in Table 1. It included 21 men and 18 women, with ages of diagnosis between 20–79 (mean age of diagnosis of 53.74 ± 12.13). All families had a positive cancer family history, except for SPS.8. Importantly, nine families had a positive family history for CRC.

### 2.2. Whole Exome Sequencing Analysis

Germline WES was performed for the entire SPS cohort including 39 samples, and a three-step prioritization workflow was carried out. The first phase consisted of variant filtering, based on variant impact (nonsynonymous, truncating), allele frequency (<0.1%) and pathogenicity prediction tools for missense variants (score > 3), identifying 393 variants in 363 genes, with an average of 24.5 variants per family. Since there was still a high number of candidate variants and genes, a second-round of prioritization based on manual curation of biological function was performed. A total of 71 variants in 69 genes with a mean of 4.5 variants per family remained (Supplementary Table S1). Finally, using more stringent filtering criteria based on compatible biological functions as epigenetic regulators, senescence and the previous involvement in cancer development, 12 candidate genes with potentially pathogenic genetic variants were selected, including *ANXA10, ASXL1, CFTR, DOT1L, INO80, KLF3, MCM3AP, MCM8, POLD1, TP53BP1, WNK2* and *WRN*. No relevant copy number variants (CNV) were identified, therefore, only single-nucleotide variants (SNV) and insertion/deletion variants (indels) were considered for variant prioritization. Final candidate variants and genes were validated by manual inspection of sequencing data and Sanger sequencing (Supplementary Figure S1A).

Somatic WES was performed in at least one somatic sample per family, except for family SPS.10 with no available samples, and SPS.5 that did not pass quality control. In one family, the available somatic sample in the family was unpaired with the corresponding germline sample (SPS.16).

### 2.3. Germline-Somatic Paired Analysis

In order to identify genes that could comply with the Knudson two-hit hypothesis for tumor-suppressor gene (TSG) inactivation, an integration of paired germline and somatic WES data was carried out. Second mutational hits could be SNVs, indels, CNVs, loss of heterozygosis (LOH) or epigenetics events. In this work, SNVs, indels, CNVs and somatic LOH were analyzed.

Five genes in family SPS.7 contained possibly pathogenic SNVs in both germline and somatic DNA (Supplementary Table S2), and *MCM3AP* stood out among them. No other two-hit SNVs were identified in other families.

Following the same germline vs. somatic WES strategy, 29 germline variants in 27 genes were identified as being affected by a second-hit LOH in the paired somatic sample (Supplementary Table S3). Among them, there were two of the previously selected candidate genes by the germline WES-only strategy (*CFTR*, *KLF3*). After manual curation for biological functions involved in cancer development, both *HIC1* (family SPS.14) and *PDLIM2* (family SPS.7) were selected as final candidate genes and further validated by Sanger sequencing (Supplementary Figure S1B).

Taking together all the different strategies to identify new candidate genes for SPS germline predisposition, including searching for potentially disruptive germline variants and germinal-tumor comparison, the final most promising candidate genes identified were *ANXA10*, *ASXL1*, *CFTR*, *DOT1L*, *HIC1, INO80, KLF3, MCM3AP, MCM8, PDLIM2, POLD1, TP53BP1, WNK2* and *WRN* (Table 2).

### 2.4. Somatic Mutational Profile

Fourteen somatic WES samples were assessed in order to characterize the somatic mutational profile of the SPS cohort. Mutational signatures, tumor mutational burden (TMB), tumor indel burden, and variants in cancer driver genes (*BRAF, KRAS, POLE, POLD1* and DNA mismatch repair (MMR) genes) were analyzed for available samples.

**Figure 2:**
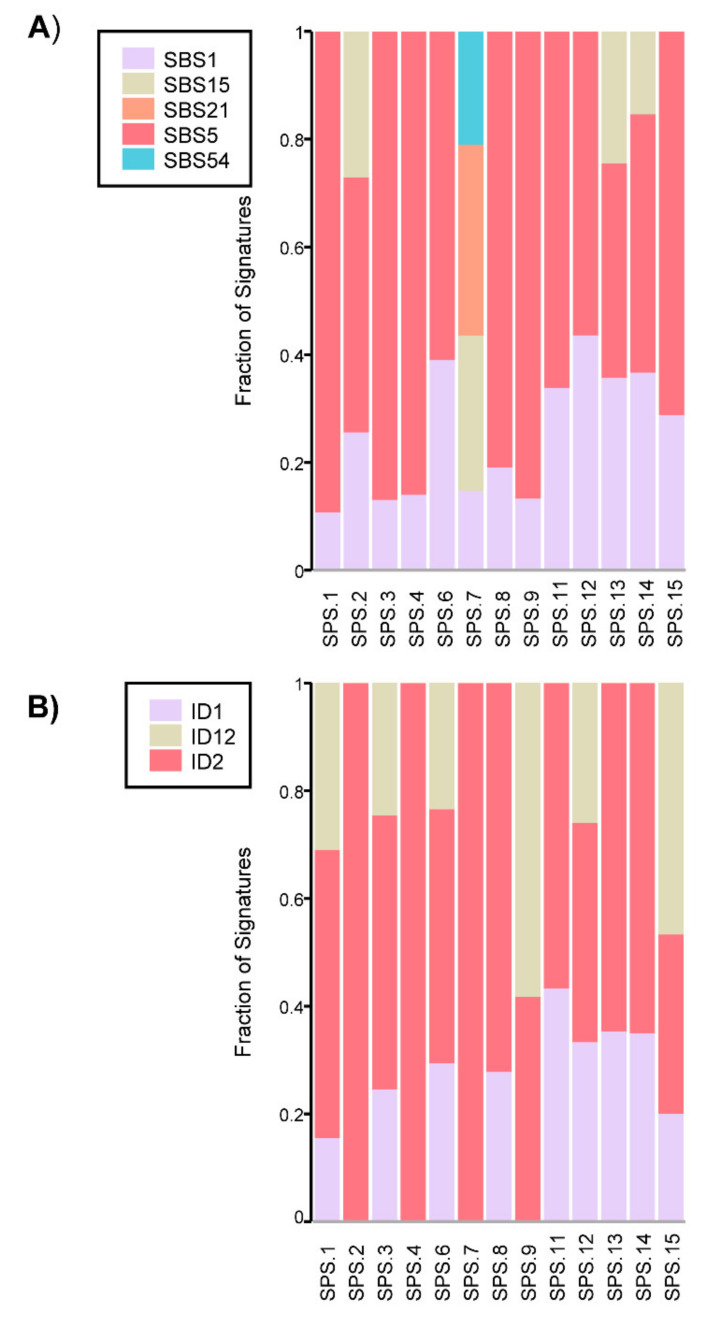
(**A**) Contribution of single base substitution (SBS) mutational signatures identified per sample. SBS1 and SBS5 are clock-like signatures. SBS15 and SBS21 are related to DNA MMR deficiency and SBS54 constitutes a sequencing artifact (https://cancer.sanger.ac.uk/cosmic/signatures/SBS/index.tt (accessed on 12 June 2020)). (**B**) Contribution of small insertions and deletions (ID) mutational signatures identified per sample. ID1 and ID2 are clock-like and DNA MMR deficiency related. ID12 has an unknown aetiology (https://cancer.sanger.ac.uk/cosmic/signatures/ID/index.tt (accessed on 12 June 2020)).

The characterization of the mutational profile included the analysis of mutational signatures, which are unique combinations of mutational footprints on the tumor left by different mutational processes. Considering the single base substitutions (SBS) mutational signatures analyzed, SBS1 and SBS5 were the most represented throughout the cohort (Figure 2A). SBS1 and SBS5 are considered clock-like mutational signatures, as they correlate with age. This means that a higher mutation rate is observed within a later age of diagnosis. This process is considered to happen in a constant rate throughout life. Other SBS signatures were also present in somatic samples of patients, such as SBS15 and SBS21. They are two of the mutational signatures related to DNA MMR deficiency and were observed in the somatic samples of families SPS.2 and SPS.7, which was concordant with the MLH1-/PMS2- tumor status in SPS.7. SBS15 alone was also present in SPS.13 and SPS.14, though, in this case, it probably represents an overfitting of this signature for these samples, since there was only a small contribution of mutations to this signature (Figure 2A). Finally, SBS54 also appeared in SPS.7, and although usually indicates some germline sample contamination, it has a tendency to come out in samples with microsatellite instability (MSI), as it is the case.

Small insertion and deletion (ID) signatures were also considered for the cohort. ID1 and ID2 had the major contribution along the samples analyzed (Figure 2B). They are found in most cancer samples and are also aging-related, since they can correlate with age of diagnosis in non-hypermutated cancer samples. However, both signatures are often elevated in cancer samples with defective DNA MMR. Overall, clock-like mutational signatures were the major contributors in our cohort with some relevance also of the DNA MMR signatures in some samples. Regarding SPS.16, with an unpaired germline sample available, SBS and ID analysis revealed prevalence of aging signatures and also presence of a MMR-deficiency mutational signature. Again, the MMR defective signature was concordant with the MLH1-/PMS2- tumor status in SPS.16.

**Figure 3:**
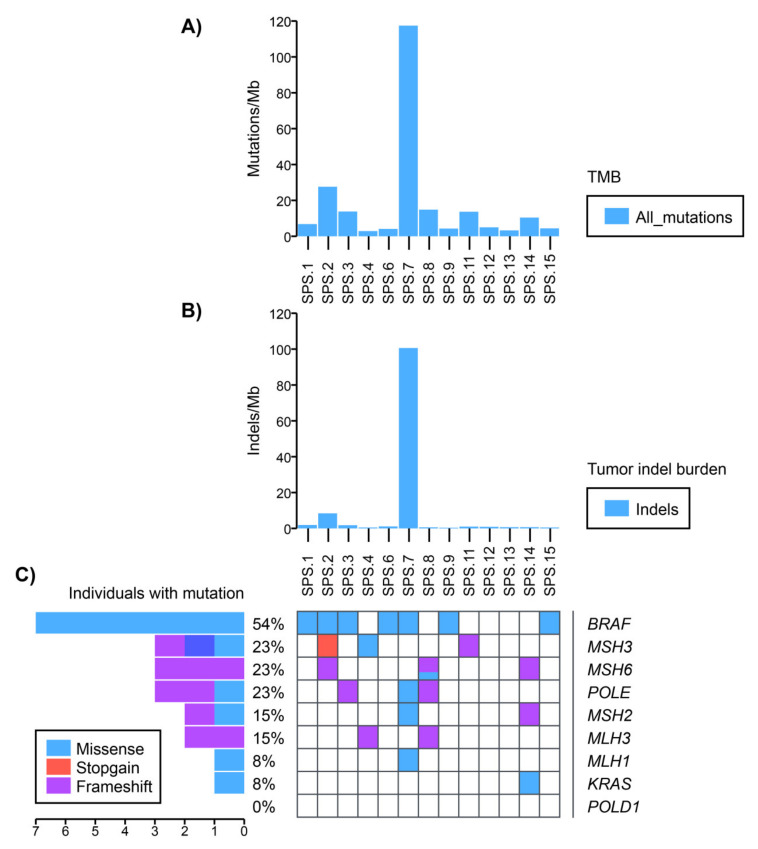
Somatic mutational profile of the SPS cohort. (**A**) Tumor mutational burden (TMB) per sample. (**B**) Tumor indel burden per sample. (**C**) Most frequent mutated cancer driver genes in each sample. Identified mutations in the most relevant cancer driver genes such as *POLE*/*POLD1*, MMR genes (*MLH1*, *MLH3*, *MSH2*, *MSH3*, *MSH6*, *PMS2*), *BRAF* and *KRAS* were analyzed in the individuals with somatic WES without using the AAF threshold of 20%.

Regarding TMB, six samples showed a hypermutated profile (>10 mutations/Mb) (families SPS.2, SPS.3, SPS.7, SPS.8, SPS.11 and SPS.14), and one sample (family SPS.7) stood out as ultra-hypermutated (>100 mutations/Mb) (Figure 3A). The whole cohort had a mean of 17.57±30.81 mutations/Mb. Among the hypermutated samples, SPS.2 was the one with the highest TMB (27.6 mutations/Mb) (Figure 3A). The unpaired tumor sample in SPS.16 also showed a hypermutated profile (89 mutations/Mb).

Interestingly, the same pattern was seen in the somatic indel burden analysis. We observed that in family SPS.7, the somatic profile was also ultra-hypermutated (100.6 indels/Mb) and that the second on the ranking was also from family SPS.2 (8.4 indels/Mb) (Figure 3B).

It is noteworthy that samples more hypermutated in the TMB or indel burden analysis (SPS.2, SPS.7, SPS.16) were the ones where MMR deficiency signatures were more evident. Also, ID1 and ID2 signatures, besides the clock-like behavior in non-hypermutated samples, were representative of MMR deficiency when the sample was hypermutated.

Cancer driver genes as *BRAF* and *KRAS* are usually found mutated in serrated polyps, along with MSI and CIMP (CpG island methylator phenotype) [5]. The analysis of somatic mutations by WES can be challenging, since the detection of variants depends on the tumor/somatic purity and is conditioned by intra-tissue heterogeneity. Considering this fact, alternative allele frequency (AAF) can vary to very low to more than 50% in case of homozygous variants. Therefore, a threshold of 20% AAF was established as a confidence value to determine somatic variants in this cohort.

Among the cancer driver genes analyzed, *BRAF* V600E was the most represented somatic mutation throughout the cohort (Figure 3C). This mutation was present in 4 out of 13 samples sequenced with AAF > 20% (families SPS.2, SPS.6, SPS.7 and SPS.15), but also present in other three samples with AAF < 20% (families SPS.1, SPS.3 and SPS.9) (Supplementary Table S4). On the other hand, only one *KRAS* (A146T) variant of unknown significance (VUS) was identified in the somatic profile of one patient in family SPS.14 (Supplementary Table S4).

Loss of MMR proteins such as MLH1 are mostly responsible for the MSI phenotype observed in serrated lesions [20]. Somatic variants in MMR genes were the second most frequent, identified in six samples within the cohort (SPS.2, SPS.4, SPS.7, SPS.8, SPS.11 and SPS.14) in the *MSH2, MSH3, MSH6, MLH1,* and *MLH3* genes (Figure 3C). Of them, one variant had AFF > 20% (*MLH1* R687Q, in SPS.7) and the other variants showed a confidence value below 20% (Supplementary Table S4).

*POLE* and *POLD1* encode for the catalytic subunits of polymerases epsilon and delta, respectively, involved in the DNA proofreading process. Mutations in these genes have been related to a hypermutator tumor phenotype [21]. Only *POLE* variants with AAF < 20% were found in somatic samples from families SPS.3, SPS.7 and SPS.8. No somatic *POLD1* variants were detected.

Hypermutated somatic profiles are mainly related to mutations in the MMR genes or *POLE* and *POLD1*, either in somatic or germline DNA. Interestingly, the ultra-hypermutated sample SPS.7 displayed the somatic R647Q *MLH1* variant, possibly explaining part of the hypermutated profile of the tumor. On the other hand, a germline variant in *POLD1* was also identified in this patient and segregated correctly in the family (Supplementary Table S1), being another possible explanation for the ultra-hypermutated somatic profile observed. Furthermore, SPS.7 harbored a somatic VUS in *MLH1* and a likely pathogenic *MSH2* mutation, possibly explaining the origin the mutational signatures SBS15 and SBS21 (Supplementary Table S4). Nevertheless, for family SPS.2, only somatic variants at very low AAF in MMR genes (*MSH3* and *MSH6*) were identified (Supplementary Table S4).

All relevant mutations identified in *BRAF, KRAS, POLE, POLD1* and MMR genes are described in Supplementary Table S4.

## 3. Discussion

SPS is a clinical condition with familial aggregation in some cases and an important risk for CRC. However, it is probably largely underdiagnosed, and the underlying germline causes are still mostly unknown. In order to identify new candidate genes for germline SPS predisposition, this study analyzed 39 SPS patients in 16 families that underwent germline and somatic WES. Following germline and paired germline-somatic analysis strategies, we suggest 14 new candidate genes to be associated with this condition including *ANXA10, ASXL1, CFTR, DOT1L, HIC1, INO80, KLF3, MCM3AP, MCM8, PDLIM2, POLD1, TP53BP1, WNK2* and *WRN*.

Senescence plays an important role in tumor progression through the serrated pathway of CRC carcinogenesis. After oncogene activation of *BRAF* or *KRAS*, the proliferation of intestinal crypt cells is initially blocked by a senescence-associated growth arrest. However, hypermethylation of *p16* and *IGFBP7* promoters leads to the surpassing of this senescence barrier, driving the progression of hyperplastic polyps to sessile serrated adenoma/polyps (SSA/P) [20]. Of note, Gala et al. reported that an enrichment of rare germline loss-of-function mutations impairing senescence-related genes was associated with the development of SSAs. Particularly, genes involved in senescence are mainly involved in p16-Rb and ATM-ATR DNA damage response pathways [11].

In this study, we identified rare, potentially pathogenic germline variants in four genes involved in senescence processes (*INO80, TP53BP1*, *WRN* and *DOT1L*). Chromatin-remodeling ATPase INO80 acts as part of the INO80 (inositol requiring mutant 80) chromatin remodeling complex, and it is involved in transcriptional regulation, DNA replication and DNA repair [22]. Min et al. demonstrated that in mouse embryo fibroblasts, depletion of Ino80 led to an activation of p21-dependent cellular senescence and impacted in DNA damage signaling and repair. p53BP1 (TP53-binding protein 1) is a key protein involved in double-strand breaks (DSBs) repair, cell cycle and apoptosis by interacting with the ATM-CHK2-P53 pathway. In CRC cells, TP53BP1 loss produced proliferation, cell apoptosis and cellular arrest [23]. *WRN* encodes for the Werner syndrome protein, a member of the RecQ helicase family protein, which harbors exonuclease and 3′ to 5′ helicase activities. It is involved in DNA replication, recombination and repair, and also plays an important role in maintaining genomic stability. Depletion of WRN is known to lead to genomic instability and premature senescence [24].

Epigenetic alterations are also an important hallmark in the serrated carcinogenesis process. For instance, DNA methylation is frequently found in early serrated lesions, either as hypermethylation of the *p16* and *IGFBP7* promoters as mentioned beforehand, or as hypermethylation of *MLH1* or *MGMT* in the SPS malignancy processes. The former is usually related with an MSI and CIMP-high phenotype, and the latter with a microsatellite-stable and CIMP-low phenotype [20]. In addition, aberrant DNA methylation may also affect the expression of TSG, contributing to the carcinogenesis process. Two candidate genes, *DOT1L*, a methyltransferase of H3K79me (also a senescence candidate gene) and *MCM3AP* were classified as epigenetic regulators. *DOT1L* (disruptor of telomeric silencing 1-like) encodes for a methyltransferase responsible for mono-, di-, and tri- methylation of lysine 79 of histone H (H3K79me), an important factor to regulate cell cycle progression at G1/S transition and DNA damage response. In human cell lines, DOT1L depletion leads to upregulation of CDK inhibitors, conducting cells to an irreversible G1 arrest [25]. In addition, DOT1L also regulates the phosphorylation of the histone variant H2AX (γH2AX) and participates in DNA repair of DSBs via homologous recombination [26]. Regarding MCM3AP (MCM3-associated protein), it binds and acetylates the MCM3 protein, negatively regulating DNA replication and cell cycle progression [27].

Finally, genes previously associated with cancer development were also prioritized. Particularly, *ASXL1, CFTR, HIC1, KLF3, MCM8, PDLIM2, POLD1* and *WNK2* were selected. *ASXL1* (Additional sex comb like 1) is a TSG involved mainly in hematological cancers, but also in other malignant processes such as breast cancer and prostate cancer. It forms a complex with BAP1 that regulates H2A deubiquitination, avoiding extrinsic and intrinsic anti-proliferative signals. Therefore, mutations in this gene may confer proliferative advantage to malignant cells [28]. *CFTR* (cystic fibrosis transmembrane conductance regulator) is well-known to be associated with cystic fibrosis (CF). The risk of CRC in adults with CF is 5–10 times greater compared to the general population and it is estimated that 25% of patients with CF will develop advanced adenomas, with a risk also extended to carriers of *CFTR* mutations [29]. The mechanisms by which *CFTR* acts as a TSG are not well understood, but its promoter methylation might trigger its downregulation in CRC [30]. *HIC1* (Hypermethylated in cancer 1) is a TSG located within a CpG island, and its methylation and loss-of-heterozygosity are very common in tumors. Traditionally, HIC1 is suggested to be related with its transcriptional repressor role and the upregulation of *SIRT1* and consequent inactivation of p53, stimulating tumor growth [31]. On the other hand, new evidence suggests HIC1 has also a relevant role in maintaining the genomic stability through the regulation of DNA repair. *Hic1* inactivation in mice cells was shown to lead to cell cycle arrest, premature senescence, chromosomal instability and spontaneous transformation in vitro [32]. KLF3 (Krüppel-like factor 3) acts as a transcriptional repressor, regulating gene expression in several biological processes such as erythropoiesis and B cell development. It has been shown to be involved in more aggressive forms of some cancers, as CRC and metastatic processes in uterine cervical cancer and sarcoma [33]. *MCM8* (mini-chromosome maintenance 8 homologous recombination repair factor) germline mutations were recently associated with early-onset Lynch like-syndrome [34]. This DNA helicase forms a complex with MCM9, both contributing to DNA repair and genomic stability. *PDLIM2* (PDZ and LIM domain 2) is downregulated in many types of NF-kB-associated tumors as leukemia, breast and colon cancer, supporting its role as a TSG in early phases of cancer development. However, it can also have a potential oncogene role later in tumorigenesis, as observed for breast cancer. Its PDZ and LIM domains are associated both with cytoskeleton and NF-kB transcription factor regulation, respectively [35]. *POLD1* (DNA polymerase delta 1) encodes for the catalytic and proofreading units of DNA polymerase δ, compromising the repair of possible DNA mismatches during DNA replication. Alteration in this function leads to an accumulation of mutations throughout the genome, potentially affecting TSGs and other cellular functions. Missense germline mutations in the exonuclease domain of *POLD1* were associated with colorectal multiple polyposis and CRC predisposition, as well as for endometrial cancer [36]. *WNK2* (WNK lysine deficient protein kinase 2) was seen to be down-regulated in SPS [37]. In hepatocellular carcinogenesis, *WNK2* was also shown to be a driver and a risk factor for early recurrence. As a serine/threonine kinase, it participates in the ERK/MAPK-pathway, regulating many cellular processes as cell signaling and cell proliferation. Finally, *ANXA10* (Annexin A10) has also demonstrated to be an interesting candidate since its expression is used as a histological marker for serrated polyps and serrated pathway to carcinogenesis. It was demonstrated that a higher *ANXA10* expression indicates high risk for developing a metachronous serrated neoplasm. *ANXA10* seems to be implicated in many biological processes as growth regulation, apoptosis and cell differentiation, although its specific function in CRC is still not fully understood [38].

Regarding previous studies, some attempts to identify rare, high-penetrance germline predisposition to SPS have been performed, including the screening of patients with family aggregation for SPS or those severely affected patients, or looking at the enrichment of specific pathways as senescence-related [11,39,40]. Currently, the only gene suggested to be involved in the germline predisposition to SPS is *RNF43*, although it seems to be involved in only some rare cases [12,13]. None of the genes suggested for the germline SPS predisposition in this study was previously observed. *RNF43* was not observed in our cohort, corroborating other studies that claim *RNF43* to be a very rare contributor to SPS [12,13].

Lastly, performing WES on somatic samples has provided insights over the mutational profile of the serrated lesions in the evaluated SPS cohort. Clock-like mutational signatures were the most represented mutational signatures. Although they are found through various tissues at variable levels, those cell types with high replication rates, such as colon cells, exhibit higher “clock-like” mutation rates [41]. For instance, a high prevalence of clock-like mutational signatures, but a low prevalence of MMR and *POLE* signatures was previously observed in a familial CRC cohort [18], as seen in this study. This fact suggests that there are no specific somatic mutational patterns that correlate specifically with SPS and/or CRC or that the current knowledge in mutational signature profiling is not capable of identifying them. On the other hand, 7% (1/14) of samples were ultra-hypermutated and 50% (7/14) were hypermutated, showing higher rates than for CRC (16% [16]; 31%, [18]). Finally, we observed that *BRAF* was the most common gene mutated among samples, in agreement with previous studies, where *BRAF* mutations were very frequent in SSA (75%) and in mixed polyps (86%) [42]. Accordingly, Horpaopan et al. [43] detected that 50% of serrated polyps presented *BRAF* p.V600E mutation in a 41-years old female patient.

In conclusion, we suggest 14 new candidate genes to be involved in the germline predisposition to SPS (*ANXA10, ASXL1, CFTR, DOT1L, HIC1, INO80, KLF3, MCM3AP, MCM8, PDLIM2, POLD1, TP53BP1, WNK2* and *WRN*) involved in senescence, epigenetics regulation or cancer. Variants detected in these genes were present in all affected members of the analyzed families, they were potentially pathogenic (truncating or with >3 pathogenicity predictions for missense variants), and a two-hit inactivation was evident for some of them. The somatic analysis of the serrated lesions in our cohort showed a relevant hypermutated profile and high contribution of clock-like mutational processes along with MMR signatures in some samples. In this sense, although SPS is a condition that might predispose to CRC, it presents its own features, and it differentiates from sporadic and the traditional adenoma-carcinoma CRC. Nevertheless, we are aware that additional replication of these results in larger cohorts and functional studies are needed to corroborate the germline predisposition involvement of these genes on SPS.

## 4. Materials and Methods

### 4.1. Patients

Thirty-nine SPS patients from 16 families with aggregation for this disease were selected in the study. *MUTYH, APC* and the DNA MMR genes were previously screened with negative results. Families were recruited on high-risk CRC clinics at Hospital Clínic de Barcelona, Hospital Donostia, Complexo Hospitalario Universitario de Ourense, and Hospital Universitario de Móstoles in Spain. Patients were diagnosed with SPS based on the criteria recommended by the WHO. Recently, the diagnostic criteria were updated excluding criteria 2. However, this study used the 3 criteria-based diagnostics.

The whole cohort underwent germline WES and at least one individual per family had a somatic sample paired sequenced when possible, seeking for tumor samples (in SPS.7, SPS.11 and SPS.14) or the most advanced serrated lesion available in the rest of families. Immunohistochemistry for *MLH1, MSH2, MSH6* and *PMS2* was performed to check MMR protein status in the tumor/somatic sample available for some families. Germline DNA samples were extracted using the QIAamp DNA Blood kit (Qiagen, Redwood City, CA, USA), whereas tumor/somatic DNA samples were obtained from formalin-fixed paraffin-embedded tissue using the QIAamp Tissue kit (Qiagen, Redwood City, CA, USA), in both cases following the manufacturer’s instructions.

### 4.2. Whole Exome Sequencing

Sequencing raw data analysis and variant filtering was performed as previously described [18,44]. Briefly, WES was performed using the HiSeq2000 Platform (Illumina, San Diego, USA) in both germline and tumor/somatic samples. For exon enrichment, the Sure SelectXT All Exon v5 kit (Agilent, Santa Clara, CA, USA) was used for both cases. Library quality control was performed with Bioanalyzer 2000 (Agilent, Santa Clara, CA, USA). Samples were indexed with adapters containing different barcodes, allowing to be pooled and massive parallel sequenced using a paired-end 2 × 100 (germline) or 2 × 75 (somatic) base pair read length protocol.

### 4.3. Sequencing Quality Control and Alignment

The post sequencing quality control used the Real-Time Analysis software sequence pipeline (Illumina, San Diego, CA, USA) before data analysis. Mean coverage was >95x in all samples. Only regions with a coverage >10x were used for the following analyses. A good ratio of shared regions with high coverage (70%) was expected in good-quality somatic samples, whereas low-quality ones were characterized by a significant drop in this percentatge and excluded. Germline WES data was mapped to the human genome hg19/GRCh37 using Genome Multitool [45], whereas the Burrows-Wheeler Aligner (BWA-MEM algorithm) was used for somatic data [46]. In both cases, PCR duplicates were processed using Picard (Broad Institute, Cambridge, USA; http://broadinstitute.github.io/picard/ (accessed on 10 June 2016)) and local indel realignment and base quality score recalibration were performed using the Genome Analysis Toolkit (GATK, Broad Institute, Cambridge, USA; https://gatk.broadinstitute.org/hc/en-us (accessed on 12 June 2020)).

### 4.4. Variant Calling

Variant calling of SNVs and indels was performed with GATK tools (Genome Analyzer Toolkit) Haplotyple Caller for germline data or with MuTect2 for somatic data. An extra somatic variant caller, SMuFin (http://cg.bsc.es/smufin/ (accessed on 12 June 2020)) [47], was also used in order to improve the confidence of somatic variant calling. Somatic variant calling was paired with the germline counterpart except for the tumor sample available in SPS.16, which was unpaired with the germline samples sequenced in that family. In this case, we performed an unpaired analysis using a pool of 110 germline WES samples available from our in-house cohort.

### 4.5. Copy number Variation Analysis

Copy number variation analysis of germline samples were performed as previously described [48]. In summary, CoNIFER (http://conifer.sourceforge.net/ (accessed on 12 June 2020)) [49] and ExomeDepth (https://cran.r-project.org/web/packages/ExomeDepth (accessed on 12 June 2020)) [50] were used for CNV identification.

### 4.6. Variant Annotation

Variant annotation was carried out with SNPEff (https://pcingola.github.io/SnpEff/ (accessed on 12 June 2020)) [51], which can predict functional effects (synonymous, nonsynonymous, frameshift and nonsense) and functional impacts (high, low, moderate, modifier) of genetic variants. Also, multiple databases were used to annotate data, including populational allele frequency databases such as dbSNP (https://www.ncbi.nlm.nih.gov/snp/ (accessed on 12 June 2020)) [52], 1000 Genomes Project (https://www.internationalgenome.org/ (accessed on 12 June 2020)) [53], the Exome Variant Server (https://evs.gs.washington.edu/EVS/ (accessed on 12 June 2020)), the Exome Aggregation Consortium (http://exac.broadinstitute.org/ (accessed on 12 June 2020)) [54], gnomAD (https://gnomad.broadinstitute.org/ (accessed on 12 June 2020)) [55] and 100 whole genomes of Spanish Ancestry from Centre Nacional d’Anàlisi Genòmica (http://www.cnag.cat (accessed on 12 June 2020)). Annotation was complemented by SNPSift [51] using the dbNSFP database [56,57] using six pathogenicity predictor tools (PhyloP, SIFT, Polyphen, MutationTaster, GERP, LRT). Biological functions and pathways of the genes containing variants were also annotated with terms and previous bibliography according to NCBI Gene (http://www.ncbi.nlm.nih.gov/gene (accessed on 12 June 2020)), Gene Ontology (http://www.geneontology.org/GO (accessed on 12 June 2020)) [58,59], KEGG (http://www.genome.jp/kegg/ (accessed on 12 June 2020)) [60], and Reactome (http://www.reactome.org/PathwayBrowser/ (accessed on 12 June 2020)) [61].

### 4.7. Variant Prioritization

It is important to highlight that in the variant prioritization strategy, an autosomal dominant inheritance pattern was mainly expected for the SPS analyzed families. However, both dominant and recessive variants were considered. It should be noted that no relevant autosomal recessive variants were detected, and therefore only heterozygous genetic variants were further selected. Also, only shared variants between all affected members sequenced in the family were considered. All previous annotated information was used to establish stringent criteria to select potentially pathogenic variants in a three-round filter selection. Firstly, candidate variants were filtered so that only variants with a MAF < 0.1% along with a nonsynonymous and/or with truncating effect were selected. In addition, it was required that missense variants fulfilled at least 3 pathogenic predictions with the bioinformatic tools used.

In a second phase of variant prioritization, a manual curation based on biological function was carried out, using as exclusion criteria if genes were previously involved in a non-cancer disease, a gene function non compatible with cancer predisposition, incomplete or vague information for gene function, incorrect segregation of the same variant in another SPS family of this study, and incorrect segregation of the same variant in our CRC cohort [18,44]. In a third-round prioritization, genes with specific biological functions compatible with cancer development, senescence and methylation were prioritized since they were shown to be relevant for SPS [11,20].

Regarding the tumor data, additional filters as a minimum germline-somatic coverage of 10x and a minimum alternative allele frequency (AAF) in the tumor of 20% was required. Identified mutations in the most relevant cancer driver genes such as *POLE*/*POLD1*, MMR genes (*MLH1*, *MLH3*, *MSH2*, *MSH3*, *MSH6*, *PMS2*), *BRAF* and *KRAS* were analyzed in the individuals with somatic WES without using the AAF threshold of 20%.

### 4.8. Variant Validation

Final candidate variants identified by WES were validated both using IGV (Integrative Genome Viewer; http://software.broadinstitute.org/software/igv/ (accessed on 12 June 2020)) [62] followed by Sanger sequencing (Eurofins Genomics, Luxembourg, Germany).

### 4.9. Somatic Data Analysis: LOH Analysis, Mutational Profiling and Mutational Signature Analysis

Given the availability of germline and somatic data, the comparison between both data following the Knudson two-hit hypothesis was performed. Therefore, one of the strategies of this study was to search for genes with potentially pathogenic events compatible with a TSG model (SNV, indels or LOH) as previously described for CRC [18].

ALFRED (allelic loss featuring rare damaging) approach was used to test Knudson’s two-hit hypothesis genome-wide, predicting the LOH of the wild-type allele in tumor/somatic samples [63].

In addition, the mutational aspects from somatic available samples from families (one sample per family) were analyzed in order to complement and support analysis of germline data, helping to shed light into the inherited predisposition in the family. Part of the analysis included evaluation of the mutational burden and mutational signatures using SigProfilerExtractor [64]. The tumors/advanced serrated polyps were then classified as hypermutated (>10 mut/megabase) or ultra-hypermutated (>100 mut/megabase). In addition, more than 60 available COSMIC database mutational signatures (https://cancer.sanger.ac.uk/cosmic/signatures (accessed on 12 June 2020)) were estimated for each somatic sample based on its mutational burden.

## 5. Conclusions

The present study brings new insights to the high penetrance germline predisposition to SPS. Although its limitations (sample size, need for replication in independent cohorts and functional studies), an in-deep, integrative analysis was carried out using both germline and somatic sequencing data. To our knowledge, this is the largest cohort of SPS assessed by this manner. Since not predominant candidate germline alteration was detected, we can corroborate the genetic heterogeneity of this clinical entity. Also, since alterations in previous hereditary polyposis genes either with a dominant or recessive inheritance were not detected, it suggests a differentiated profile for it. Based on our integrated analysis, we suggest 14 genes as candidates to participate in the germline predisposition to SPS including *ANXA10, ASXL1, CFTR, DOT1L, HIC1, INO80, KLF3, MCM3AP, MCM8, PDLIM2, POLD1, TP53BP1, WNK2* and *WRN*.

## Figures and Tables

**Table 1:** Clinical characteristics of serrated polyposis syndrome families. Thirty-nine SPS patients from 16 families (≥2 patients per family) were recruited in the study. Age at diagnosis is specified.

Family	ID	Onset Age	Gender	Diagnosis	Criteria SPS	CRC	MMR +/−	Family Cancer History
SPS.1	AA3519 *	58	M	SPS	1 + 3	N	N/A	Prostate (father, 82)
	AA3520	62	F	SPS	2	N		
SPS.2	AA3521 *	62	M	SPS	1 + 3	N	N/A	Pancreas (father, 59), Colon (mother, 78)
	AA3522	59	M	SPS	2	N		
SPS.3	AA3523	32	M	CRC	-	Y	+	CRC (mom, 56), CRC (brother, 56), Pancreas (aunt, 60), CRC (uncle, 53), CRC (uncle, 71), CRC (cousin, 32), Brain (cousin, 32)
	AA3524 *	64	F	SPS	3	N		
SPS.4	AA3525	58	M	SPS	3	N	+	CRC (father, 86), Laryngeal (father, 85), Breast (aunt, 61), Breast (aunt, 56), CRC (grandfather, 61)
	AA3526 *	53	F	SPS	2	N		
SPS.5	AA3527	22	F	SPS	3	N	N/A	Laryngeal (grandfather, unknown age)
	AA3528 *	20	M	SPS	2	N		
SPS.6	AA3529	61	F	SPS	3	N	+	CRC (sister, 43), CRC (aunt, 70), Gastrointestinal (father, 54)
	AA3530 *	53	F	SPS	2	N		
SPS.7	AA3531 *	57	F	SPS	1 + 3	Y	MLH1-/	Lymphoma (sister, 46), Gastric (grandfather, 48)
	AA3532	56	M	SPS	2	N	PMS2-	
SPS.8	AA3533 *	62	M	SPS	3	N	N/A	-
	AA3534	65	M	SPS	2	N		
SPS.9	AA3535	65	F	SPS/CRC	2 + 3	Y	N/A	CRC (father, 62)
	AA3536	70	F	SPS/mixed polyposis	2	N		
	AA3537 *	64	M	Mixed polyposis	3	N		
SPS.10	AA3538	58	F	CRC	-	Y	+	CRC (mother, 58), Lung (mother, 70), CRC (uncle, 77), CRC (grandfather, 83), Thyroid (sister, 57), Skin (sister, 42), Liver (cousin, 28), Liver (cousin, unknown age), Skin (grandmother, unknown age)
	AA3539	60	M	SPS	2	N		
	AA3540	53	F	SPS	2	N		
	AA3541	40	F	SPS	1 + 3	N		
SPS.11	AA3559	39	F	SPS	2	N	+	Skin (grandmother, 91), CRC (mother, 54), CRC (father, 64), Endometrial (aunt, 72), Endometrial (grandmother, 70), Ovarian teratoma (niece, 7)
	AA3560	46	F	SPS	1	N		
	AA3561	38	F	SPS	2	N		
	AA3562	44	M	SPS	2	N		
	AA3563 *	54	F	CRC	1	Y		
	AA3564	48	M	SPS/adenomas	2	N		
SPS.12	AA3565	55	M	SPS	2 + 3	N	+	CRC (sister, 46)
	AA3566 *	59	M	SPS	1 + 2 + 3	N		
SPS.13	AA3567 *	59	M	SPS	2 + 3	N	N/A	Thyroid (mother, 66), Endometrial (mother, 76), Unknown cancer (father, 75)
	AA3568	55	F	SPS	1 + 2 + 3	N		
SPS.14	AA3569 *	68	M	SPS/CRC	2 + 3	Y	N/A	CRC (uncle, 85), Pancreas (father, 75)
	AA3570	74	M	SPS	2 + 3	N		
SPS.15	AA3571	67	M	SPS	2	N	N/A	Sarcoma (father 51), Thyroid (cousin, 21), Glioma (cousin, 23)
	AA3572 *	38	M	SPS	1 + 3	N		
SPS.16	AA3573	47	F	SPS	2 + 3	N	MLH1-/PMS2-	Gastric (father, 72), Brain (mother, 71), Pancreas (uncle, 61), Lung (uncle, 67), Bile duct (uncle, unknown age), Gastric (grandfather, 61)
	AA3574	51	M	SPS	2	N		

ID, identification; M, male; F, female; SPS, serrated polyposis syndrome; MMR, mismatch repair; N/A, non-available; Y, yes; N, no; CRC, colorectal cancer; prostate, pancreas, brain, laryngeal, breast, gastrointestinal, lung, thyroid, liver, skin, endometrial, bile duct and gastric refer to the type of cancers. * Individuals with a somatic sample analyzed by WES with any cancer listed with their respective age of onset. In MMR +/−, results for immunochemistry analysis for MMR proteins (*MLH1*, *MSH2*, *MSH6* and *PMS2*) are shown. In family cancer history, affected relatives are indicated as follows: type of cancer (relative, age at diagnosis).

**Table 2:** Candidate genes to the germline predisposition to serrated polyposis syndrome after final prioritization based on germline only and germline-somatic paired analysis. Candidate genes to the germline predisposition to SPS after final prioritization based on germline only and germline-somatic paired analysis.

Gene	Family	Variant	Pred. Tools	gnomAD	Protein Domain Location	Biological Process	Analysis Approach	Selection Criteria
*ANXA10*	SPS.12	c.692A>G p.(Asp231Gly)	6	0.000036	Annexin 3 (171–243 AA)	growth regulation; apoptosis; cell division	G	Histological marker for serrated pathway
*ASXL1*	SPS.13	c.2110G>A p.(Gly704Arg)	6	0.000668	No	transcription; cell morphogenesis; hemopoiesis; homeostasis of number of cells; protein deubiquitination;	G	Involvement in cancer development
*CFTR*	SPS.3	c.3995C>A p.(Pro1332His)	6	N/A	Nucleotide -Binding 2 (1207–1436 AA)	transmembrane transport; positive regulation of voltage-gated chloride channel activity; protein deubiquitination;	G	Involvement in cancer development
	SPS.14	c.1601C>A p.(Ala534Glu)	3	0.0000159	Nucleotide -Binding 1 (390–645 AA)			
*DOT1L*	SPS.11	c.3307G>A p.(Val1103Met)	5	0.0000349	No	chromatin silencing; histone H3-K79 methylation; regulation of JAK-STAT cascade; telomere organization; DNA repair	G	Senescence and epigenetics regulator candidate
*HIC1*	SPS.14	c.1295A>G p.(Gln432Arg)	3	N/A	Zinc finger 1 (439–459 AA)	regulation of transcription; positive regulation of DNA damage response, signal transduction by p53 class mediator	G-S paired analysis	Involvement in cancer development
*INO80*	SPS.6	c.4271G>C p.(Arg1424Pro)	5	0.00022	No	mitotic sister chromatid segregation; double-strand break repair via homologous recombination	G	Senescence candidate
*KLF3*	SPS.11	c.275C>T p.(Ser92Leu)	4	0.000203	No	regulation of transcription; cellular response to peptide	G	Involvement in cancer development
*MCM3AP*	SPS.4	c.1592A>G p.(Glu531Gly)	4	0.000502	No	mRNA transport; nucleosome organization; somatic hypermutation of immunoglobulin genes	G	Epigenetics regulator candidate
	SPS.7	c.5327T>C p.(Val1776Ala)	6	0.000191	No			
*MCM8*	SPS.6	c.876-1delG	N/A	N/A	No	G1/S transition of mitotic cell cycle; mitotic cell cycle; double-strand break repair via homologous	G	Involvement in cancer development
*PDLIM2*	SPS.7	p.(Pro130Leu)	3	N/A	No	stability of Nuclear Factor kappa-B (NFκB) and other transcription factors; polarized cell migration	G-S paired analysis	Involvement in cancer development
*POLD1*	SPS.7	c.1941delG p.(Lys648fs)	N/A	N/A	Polymerase domain (581–910 AA)	mitotic cell cycle; DNA replication; DNA repair; DNA replication proofreading; DNA damage response	G	Involvement in cancer development
*TP53BP1*	SPS.13	c.3835G>A p.(Glu1279Lys)	6	0.000008	No	DNA repair; positive regulation of transcription; DNA damage checkpoint; protein sumoylation	G	Senescence candidate
*WNK2*	SPS.4	c.4820C>T p.(Ala1607Val)	5	0.0000788	No	protein phosphorylation; negative regulation of cell proliferation; regulation of ion homeostasis	G	Involvement in cancer development
	SPS.6	c.6157G>A p.(Val2053Ile)	5	0.0000434	No			
*WRN*	SPS.1	c.2023G>C p.(Glu675Gln)	4	0.00000795	Helicase ATP-binding (558–724 AA)	telomere maintenance: DNA synthesis involved in DNA repair; replicative cell aging; DNA metabolic process	G	Senescence candidate

Pred. tools: score given by the in-house pipeline based on missense pathogenicity prediction tools (0–6). gnomAD, genome aggregation database variant frequency. G, germline; S, somatic; N/A, Not Applicable or Not Available. AA, amino acid.

## Data Availability

The data are not publicly available due to our institutional policy.

## Supplementary Materials

The following are available online at https://www.mdpi.com/2072-6694/13/4/929/s1, Figure S1: (A) Sanger sequencing validation of germline variants identified by WES in ANXA10 (family SPS.12) and DOT1L (family SPS.11). Manual inspection using Integrative Genome Viewer (IGV) and the corresponding Sanger sequencing validation. (B) Paired germline and somatic WES analysis using ALFRED suggested PDLIM2 (family SPS.7) and HIC1 (family SPS.14) as candidate genes undergoing two-hit inactivation. Genetic variants in germline (G) and somatic (S) DNA samples were verified by IGV, and LOH was validated using Sanger sequencing in both germline and somatic samples. Table S1: Candidate variants and genes per family second round prioritization, based on post manual curation of biological function. A total of 71 variants in 68 genes were prioritized. Table S2: Two-hit SNVs identification from paired germline-somatic WES data. Table S3. Germline candidate variants and genes for predisposition to serrated polyposis syndrome identified by ALFRED analysis to present loss of heterozygosity (LOH) in the paired tumor/somatic sample. Table S4. Identified mutations in the most relevant cancer driver genes. POLE/POLD1, MMR genes (MLH1, MLH3, MSH2, MSH3, MSH6, PMS2), BRAF and KRAS were analyzed in the individuals with somatic whole-exome sequencing.
