# Supplementary material for: Germline and Somatic Whole-Exome Sequencing Identifies New Candidate Genes Involved in Familial Predisposition to Serrated Polyposis Syndrome
Source: Cancers (Basel). 2021 Feb 23;13(4):929. doi: 10.3390/cancers13040929 (PMC7927050; doi:10.3390/cancers13040929)
Supplement: Supplementary file 1 [file cancers-13-00929-s001.zip › Figure Supl 1.pdf]

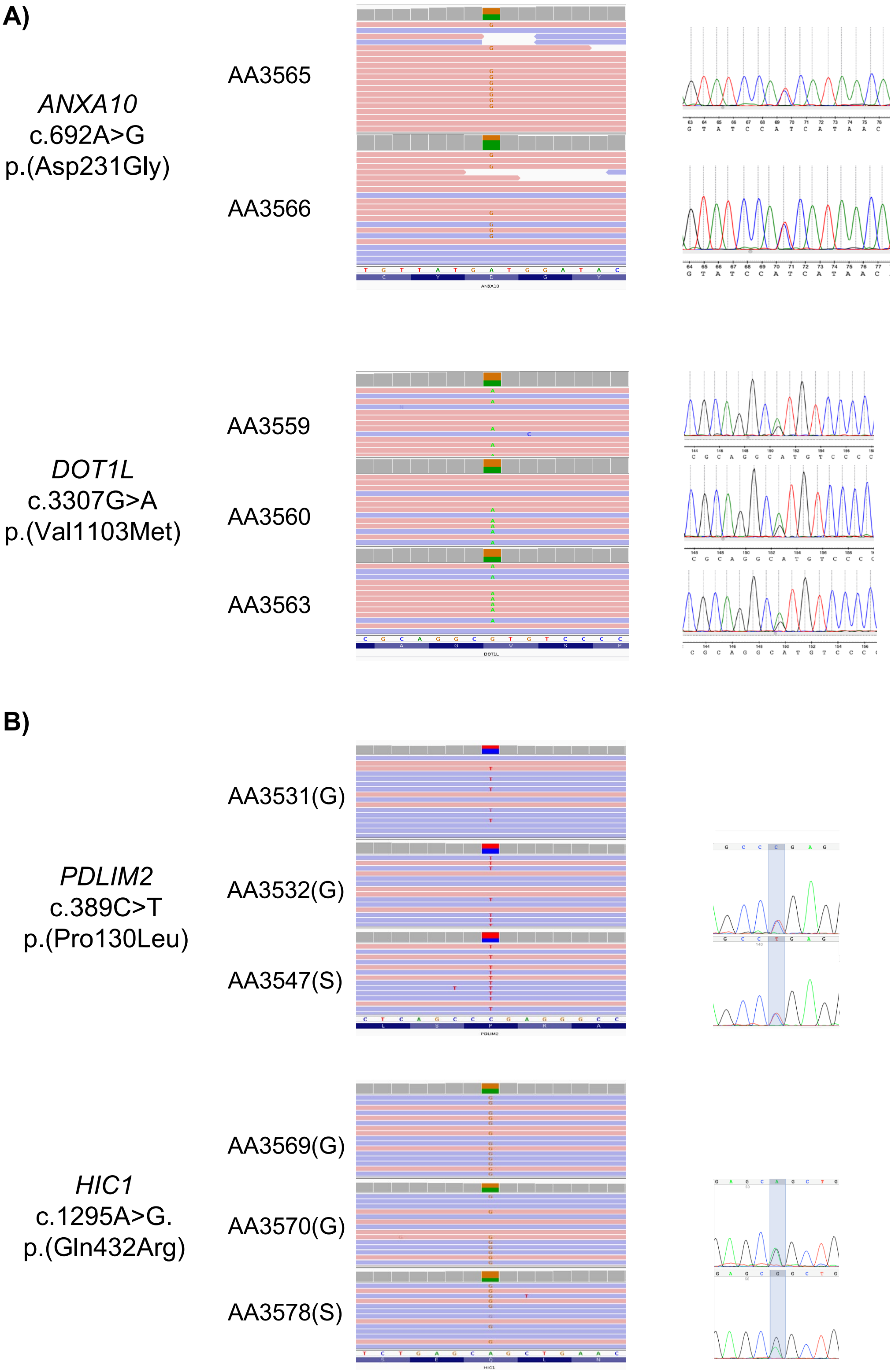
A)
ANXA10
c.692A>G
p.(Asp231Gly)
AA3565
AA3566
ANXA10
DOT1L
c.3307G>A
p.(Val1103Met)
AA3559
AA3560
AA3563
DOT1L
B)
PDLIM2
c.389C>T
p.(Pro130Leu)
AA3531(G)
AA3532(G)
AA3547(S)
PDLIM2
HIC1
c.1295A>G.
p.(Gln432Arg)
AA3569(G)
AA3570(G)
AA3578(S)
HIC1
