# Supplementary material for: Germline and Somatic Whole-Exome Sequencing Identifies New Candidate Genes Involved in Familial Predisposition to Serrated Polyposis Syndrome
Source: Cancers (Basel). 2021 Feb 23;13(4):929. doi: 10.3390/cancers13040929 (PMC7927050; doi:10.3390/cancers13040929)
Supplement: Supplementary file 1 [file cancers-13-00929-s001.zip › Soares de Lima et al_Supplementary material.pdf]

Supplementary Table 1. Candidate variants and genes selected in the second-round prioritization, based on post manual curation for biological function. A total of 71 variants in 68 genes were prioritized.

Family	Gene	Variant	Pred. tools	gnomAD	Biological Process
SPS.1	*IGFBP5*	c.769G>T p.(Val257Phe)	4	0.000365	regulation of cell growth; negative regulation of growth; glucose metabolic process; signal transduction
	LLGL1	c.2883-1G>C	N/A	N/A	protein complex assembly; exocytosis; Golgi to plasma membrane transport; axonogenesis; cortical actin cytoskeleton organization
	PPARD	c.1093A>G p.(Met365Val)	3	N/A	negative regulation of transcription from RNA polymerase II promoter; glucose metabolic process
	WRN	c c.2023G>C p.(Glu675Gln)	4	0.00000795	telomere maintenance; DNA synthesis involved in DNA repair; replicative cell aging; DNA metabolic process
SPS.2	*ABI3BP*	c.2923C>T p.(Arg975Trp)	3.25	0.000101	positive regulation of cell-substrate adhesion; extracellular matrix organization
	EWSR1	c.779A>G p.(Tyr260Cys)	4	N/A	transcription; regulation of transcription
	MTHFR	c.1097G>A p.(Arg366His)	6	0.0000177	response to hypoxia; cellular amino acid metabolic process; methionine metabolic process; one-carbon metabolic process
	NFATC1	c.860C>T(p.Pro274Leu)	6	0.000277	G1/S transition of mitotic cell cycle; epithelial to mesenchymal transition; transcription from RNA polymerase
	SCUBE2	c.2873T>A p.(Leu958*)	4.25	N/A	multicellular organism development
SPS.3	*CFTR*	c.3995C>A p.(Pro1332His)	6	N/A	transmembrane transport; positive regulation of voltage-gated chloride channel activity
	NPTX2	c.1211C>T p.(Pro404Leu))	5	0.0000538	chemical synaptic transmission
SPS.4	*CDH16*	c.1438G>A p.(Glu480Lys)	4	0.000219	cell adhesion; calcium-dependent cell-cell adhesion via plasma membrane cell adhesion molecules; cell-cell signaling; nervous system development
	DHX32	c.35C>T p.(Ser12Phe)	3	0.0000955	mRNA splicing, via spliceosome
	MCM3AP	c.1592A>G p.(Glu531Gly)	4	0.000502	immune system process; DNA replication; protein import into nucleus; mRNA transport
	WNK2	c.4820C>T p.(Ala1607Val)	5	0.0000788	protein phosphorylation; negative regulation of cell proliferation; regulation of ion homeostasis
SPS.5	*PKMYT1*	c.991G>A p.(Glu331Lys)	6	0.0000184	regulation of cyclin-dependent protein serine/threonine kinase activity; G1/S transition of mitotic cell
	PROX1	c.584C>T p.(Pro195Leu)	3	0.0000992	negative regulation of transcription from RNA polymerase II promoter; cell fate determination
	SMARCC1	c.560T>C p.(Ile187Thr)	5	0.0000762	chromatin organization; nucleosome disassembly; transcription; negative regulation of proteasomal ubiquitin-dependent protein catabolic process
	USP12	c.128A>G p.(Asn43Ser)	5	0.00000398	ubiquitin-dependent protein catabolic process; protein deubiquitination

Supplementary Table 1 *cont.* Candidate variants and genes selected in the second-round prioritization, based on post manual curation of biological function. A total of 71 variants in 68 genes were prioritized.

SPS.6	*GATA2*	c.121C>G p.(Pro41Ala)	3	0.000619	negative regulation of transcription from RNA polymerase II promoter; urogenital system
	HERC2	c.677A>G p.(Gln226Arg)	5	0.00000403	DNA repair; intracellular protein transport; cellular response to DNA damage
	INO80	c.4271G>C p.(Arg1424Pro)	5	0.00022	mitotic sister chromatid segregation; double-strand break repair via homologous
	MCM8	c.876-1delG	N/A	N/A	G1/S transition of mitotic cell cycle; mitotic cell cycle; double-strand break repair via homologous recombination
	WNK2	c.6157G>A p.(Val2053Ile)	5	0.0000434	protein phosphorylation; negative regulation of cell proliferation; regulation of ion homeostasis
SPS.7	*MYH11*	c.2289A>G p.(Ile763Met)	3	N/A	muscle contraction; axon guidance; metabolic process; skeletal muscle myosin thick filament assembly
		c.1913C>T p.(Ser638Leu)	4	0.0001033	
	DCC	c.824G>A p.(Arg275Gln)	3	0.000085	neuron migration; axonogenesis; apoptotic signaling pathway; regulation of nucleic acid-templated transcription
	MCM3AP	c.5327T>C p.(Val1776Ala)	6	0.000191	mRNA transport; nucleosome organization; somatic hypermutation of immunoglobulin genes
	POLD1	c.1941delG p.(Lys648fs)	N/A	N/A	mitotic cell cycle; telomere maintenance; DNA replication; DNA repair; DNA replication proofreading; DNA damage response
SPS.8	*DAB2IP*	c.1460G>A p.(Arg487Gln)	6	0.000113	negative regulation of transcription from RNA polymerase II promoter; activation of MAPKKK activity
	FAM83H	c.361G>A p.(Glu121Lys)	5	0.00000806	biomineral tissue development; intermediate filament cytoskeleton organization; positive regulation of cell migration
	GGNBP2	c.728G>T p.(Cys243Phe)	6	N/A	multicellular organismal development; spermatogenesis; cell differentiation; labyrinthine layer blood
	MTHFR	c.919C>T p.(Arg307Trp)	5	N/A	response to hypoxia; cellular amino acid metabolic process; methionine metabolic process; one-carbon metabolic process; neural tube closure
	RERGL	c.362T>C p.(Val121Ala)	6	0.000437	small GTPase mediated signal transduction
SPS.9	*MIA3*	c.4373C>T p.(Ser1458Phe)	5.25	0.0000161	chondrocyte development; exocytosis; regulation of cell adhesion; protein transport; regulation of cell migration; regulation of bone mineralization; lipoprotein transport
	TNK2	c.2342G>A p.(Arg781Gln)	5	0.00004775	endocytosis; cell surface receptor signaling pathway; phosphorylation; cell migration; cell differentiation; regulation of cell proliferation; innate immune response

Supplementary Table 1 *cont.* Candidate variants and genes selected in the second-round prioritization, based on post manual curation of biological function. A total of 71 variants in 68 genes were prioritized.

SPS.10	*THAP11*	c.35A>G p.(Tyr12Cys)	5	N/A	transcription; regulation of transcription
SPS.11	*CHAF1B*	c.946C>T p.(Arg316Cys)	5	0.0000141	reproduction; DNA replication; DNA repair; DNA replication-dependent nucleosome
	DOT1L	c.3307G>A p.(Val1103Met)	5	0.0000349	chromatin silencing; histone H3-K79 methylation; regulation of JAK-STAT cascade; telomere organization; DNA repair
	KLF3	c.275C>T p.(Ser92Leu)	4	0.000203	regulation of transcription; multicellular organism development; cellular response to peptide
	RASEF	c.1745C>A p.(Thr582Asn)	3	0.0000884	intracellular protein transport; metabolic process; Rab protein signal transduction; small GTPase mediated signal transduction
	SAFB2	c.1328G>A p.(Arg443His)	5	N/A	regulation of mRNA processing; regulation of transcription by RNA polymerase II; regulation of androgen receptor signaling pathway
SPS.12	*ANXA10*	c.692A>G p.(Asp231Gly)	6	0.000036	growth regulation; apoptosis; cell differentiation
	CDK9	c.1060A>G p.(Thr354Ala)	3	0.0000318	DNA repair; regulation of DNA repair; transcription
	PTCH2	c.3148C>T p.(Arg1050Trp)	3.25	0.0000119	signal transduction; epidermis development; negative regulation of smoothened signaling pathway; cell fate determination
	RALGDS	c.1337C>T p.(Thr446Ile)	5	N/A	Ras protein signal transduction; positive regulation of GTPase activity; neurotrophin TRK
	SNRK	c.1097A>G p.(Lys366Arg)	3	N/A	protein phosphorylation; myeloid cell differentiation; intracellular signal transduction
	VGLL4	c.709G>A p.(Val237Met)	6	0.0000884	regulation of transcription; negative regulation of *Wnt* signaling pathway; regulation of cell growth; negative regulation of cardiac muscle cell proliferation
SPS.13	*ASXL1*	c.2110G>A p.(Gly704Arg)	6	0.000668	transcription; negative regulation of fat cell differentiation; bone development; bone marrow development; cell morphogenesis; protein deubiquitination; thymus development
	BRCA2	c.4585G>A p.(Gly1529Arg)	6	0.000401	double-strand break repair; regulation of transcription; regulation of cytokinesis; oocyte maturation
	EPS8	c.1822A>C p.(Lys608Gln)	3	0.000337	Rho-protein signal transduction; sensory perception of sound; positive regulation of ruffle assembly
	PTPRT	c.808C>T p.(Arg270Cys)	4	0.0000121	protein dephosphorylation; cell adhesion; homophilic cell adhesion via plasma membrane
	TP53BP1	c.3835G>A p.(Glu1279Lys)	6	0,000008	DNA repair; positive regulation of transcription; DNA damage checkpoint; protein sumoylation

Supplementary Table 1 *cont.* Candidate variants and genes selected in the second-round prioritization, based on post manual curation of biological function. A total of 71 variants in 68 genes were prioritized.

SPS.14	*CFTR*	c.1601C>A p.(Ala534Glu)	3	0.0000159	intracellularly ATP-gated chloride channel activity
	FRS3	c.968A>G p.(Glu323Gly)	6	0.000136	signal transduction; fibroblast growth factor receptor signaling pathway; MAPK cascade; positive regulation of GTPase activity
	HIC1	c.1295A>G p.(Gln432Arg)	3	N/A	regulation of transcription; multicellular organismal development; regulation of *Wnt* signaling pathway; regulation of DNA damage response
	PIK3R4	c.1838C>T p.(Ser613Phe)	6	N/A	protein phosphorylation; protein targeting to vacuole; phospholipid metabolic process; innate immune response; regulation of cytokinesis
	PTPRD	c.2671T>C p.(Tyr891His)	5.25	0.0000283	protein dephosphorylation; phosphate-containing compound metabolic process; heterophilic cell-cell adhesion
	SMPD3	c.1360G>A p.(Val454Ile)	6	0.000301	hematopoietic progenitor cell differentiation; sphingolipid metabolic process; glycosphingolipid metabolic process; cell cycle
SPS.15	*GSTM3*	c.572G>A p.(Arg191His)	3	0.0000283	negative regulation of transcription from RNA polymerase II promoter; cell fate
	METTL13	c.1837C>G p.(Leu613Val)	5	N/A	methylation
	PAMR1	c.298G>C p.(Gly60Arg)	6	0.0000424	metabolic process
	PROX1	c.584C>T p.(Pro195Leu)	3	0.0000992	negative regulation of transcription from RNA polymerase II promoter; cell fate
	PTCH2	c.358C>T p.(Arg120Cys)	4	0.00001591	signal transduction; epidermis development; smoothened signaling pathway
	TRPM8	c.832C>T p.(Arg278Trp)	6	0.0000707	cellular calcium ion homeostasis; thermoception; calcium ion transmembrane transport
SPS.16	*DNMT3B*	c.-1_1delCA p.(Met1fs)	N/A	0.000318	negative regulation of transcription from RNA polymerase II; DNA methylation
	HERC1	c.7642G>C p.(Ala2548Pro)	3	N/A	negative regulation of autophagy; protein ubiquitination; cerebellar Purkinje cell
	PLCD1	c.1258C>T p.(Arg420Cys)	5	0.000262	angiogenesis;phospholipid metabolic process;lipid catabolic process;intracellular signal
	RB1CC1	c.4540C>T p.(Arg1514Cys)	5	0.000217	autophagic vacuole assembly;liver development;positive regulation of protein
	VWF	c.1625C>G p.(Ala542Gly)	3	0.000689	extracellular matrix organization;platelet degranulation;cell adhesion;blood coagulation

Pred. tools: score given by the in-house pipeline based on missense pathogenicity prediction tools (0-6). gnomAD, genome aggregation database variant frequency. N/A, Non-available.

Supplementary Table 2. Two-hit SNVs identification from paired germline-somatic WES data.

Family	Gene	Tissue	Variant	Pred. tools	gnomAD	Biological process
SPS.7	*PTPRS*	G	c.3487C>T p.(Arg1163Cys)	4	0.000012	neurological development; protein dephosphorylation; synapse organization
		S	c.2975C>T p.(Ala992Val)	4	0.01982	
	MCM3AP	G	c.5327T>C p.(Val1776Ala)	6	0.000191	immune system process; DNA replication; protein import into nucleus; mRNA transport
		S	c.5038+2T>C	N/A	N/A	
	CABIN1	G	c.6542C>T p.(Ser2181Phe)	6	0.0000248	DNA replication-independent nucleosome assembly; cell surface receptor signaling pathway; chromatin modification;
		S	c.358G>T p.(Asp120Tyr)	6	N/A	
	EIF4G1	G	c.2266G>A p.(Gly756Arg)	4	0.00001417	gene expression; cytokine-mediated signaling pathway; mitochondrion organization; negative regulation of autophagy; regulation cell cycle
		S	c.582A>T p.(Gln194His)	5	N/A	
		S	c.3362G>A p.(Arg1121His)	5	N/A	
	PHF19	G	c.710G>A p.(Arg237Gln)	5.25	0.0000319	regulation of transcription; chromatin modification; regulation of gene expression, epigenetic
		S	c.146G>A p.(Arg49Gln)	6	0.0000106	

Pred. tools: score given by the in-house pipeline based on missense pathogenicity prediction tools (0-6). gnomAD, genome aggregation database variant frequency. G, germinal; S, somatic; N/A, Not Available.

Supplementary Table 3. Germline candidate variants and genes for predisposition to serrated polyposis syndrome identified by ALFRED analysis to present loss of heterozygosity (LOH) in the paired tumor /somatic sample.

Family	Gene	Location	Germline variant	Pred. tools	gnomAD
SPS.1	*SGSM3*	22:40803845	c.1577G>A p.(Arg526Gln)	5	0.000264
	DOCK2	5:169496186	c.4690G>A p.(Asp1564Asn)	3	0.0000602
SPS.2	*F13A1*	6:6305670	c.233G>A p.(Arg78His)	3.75	0.0000159
	UTRN	6:144768755	c.1740G>A p.(Met580Ile)	4	0.000427
	KCNH2	7:150644718	c.1921A>G p.(Ser641Gly)	3	N/A
SPS.4	*CEP192*	18:13073117	c.5549C>G p.(Ser1850Cys)	6	0.000103
SPS.6	*ATG2B*	14:96756034	c.5965C>G p.(Leu1989Val)	5	0.000569
	TIAM1	21:32575246	c.2471C>T p.(Pro824Leu)	3	0.0000424
SPS.7	*DNM3*	1:172002257	c.701T>G p.(Val234Gly)	6	N/A
	MCM3AP	21:47662815	c.5327T>C p.(Val1776Ala)	6	0.000191
	USP18	22:18653593	c.797C>T p.(Thr266Met)	3	0.00000758
	PDLIM2	8:22442603	c.389C>T p.(Pro130Leu)	3	0.00001990
	PHF19	9:123629148	c.710G>A p.(Arg237Gln)	5.25	0.0000319
SPS.8	*TENM2*	5:167642142	c.3916T>A p.(Ser1306Thr)	5	N/A
SPS.11	*ARHGEF16*	1:3383881	c.784A>G p.(Thr262Ala)	4	0.000412
	SLCO4A1	20:61300405	c.2000T>G p.(Ile667Arg)	3	0.0000482
	KLF3	4:38690423	c.275C>T p.(Ser92Leu)	4	0.000203
SPS.12	*TXNRD2*	22:19898900	c.662C>T (p.Thr221Met)	6	0.000057
	TXNRD2	22:19898906	c.656G>C (p.Gly219Ala)	6	0.000303
SPS.13	*ADAMTS14*	10:72509652	c.2347G>A (p.Ala783Thr)	5	0.0000239
	CHD1	5:98233967	c.1358A>G p.(Asp453Gly)	3	N/A
	CD93	20:23066529	c.301A>T p.(Lys101*)	3.25	N/A
	HLA-DQA1	6:32610752	c.637G>T p.(Glu213*)	3.5	0.0000725
SPS.14	*HIC1*	17:1961279	c.1295A>G. p.(Gln432Arg)	3	N/A
	PCDH1	5:141244937	c.959A>C p.(His320Pro)	4	0.000114
	TENM2	5:167689642	c.8125G>A p.(Gly2709Arg)	6	0.0000395
	CFTR	7:117227809	c.1601C>A p.(Ala534Glu)	3	0.0000159
	C12orf55	12:96883648	c.266delA p.(Lys89fs)	N/A	N/A
SPS.15	*SKA3*	13:21732060	c.1119+1G>C	N/A	0.0002216

Pred. tools: score given by the in-house pipeline based on missense pathogenicity prediction tools (0-6). gnomAD, genome aggregation database variant frequency. N/A, Not Available

Supplementary Table 4. Identified mutations in the most relevant cancer driver genes. *POLE*/*POLD1*, *MMR* genes (*MLH1*, *MLH3*, *MSH2*, *MSH3*, *MSH6*, *PMS2*), *BRAF* and *KRAS* were analyzed in the individuals with somatic whole-exome sequencing.

Family	Gene	Mutation	AAF (%)	ClinVar
SPS.1	*BRAF*	V600E	17%	Pathogenic
SPS.2	*MSH3*	K383fs	12%	Not rep.
		Y789delins*LFIIL	2.50%	Not rep.
	MSH6	T1085fs	4%	Pathogenic
	BRAF	V600E	38%	Pathogenic
SPS.3	*POLE*	L570fs	11%	Not rep.
	BRAF	V600E	19%	Pathogenic
SPS.4	*MLH3*	S748fs	2%	Not rep.
	MSH3	T480K	7%	Not rep.
SPS.6	*BRAF*	V600E	25%	Pathogenic
SPS.7	*POLE*	H1820Y	12%	Not rep.
	MLH1	R687Q	20%	VUS
	MSH2	C333R	18%	Likely Path.
	BRAF	V600E	26%	Pathogenic
SPS.8	*POLE*	V246fs	5%	Not rep.
	MLH3	H6111fs	3%	Not rep.
		S490fs	2%	Not rep.
		S111fs	1.40%	Not rep.
	MSH6	K676Q	1.60%	Not rep.
		K311fs	4%	Not rep.
		K676fs	1.60%	Not rep.
		P982fs	2.40%	Not rep.
SPS.9	*BRAF*	V600E	9%	Pathogenic
SPS.11	*MSH3*	R938fs	4%	Not rep.
SPS.14	*MSH2*	C778fs	2%	Not rep.
	MSH6	Y730fs	2%	Not rep.
	KRAS	A146T	53%	VUS
SPS.15	*BRAF*	V600E	23%	Pathogenic

There was no somatic data available for families SPS.5 and SPS.10. SPS.16 tumor sample was unpaired with the germline counterpart so somatic variants for this sample are not shown. The table shows all mutations identified in the cancer driver. The table shows all mutations identified in the cancer driver genes regardless the AAF > 20% filter. AAF, alternative allele frequency; Not rep., not reported; Likely Path., likely pathogenic.

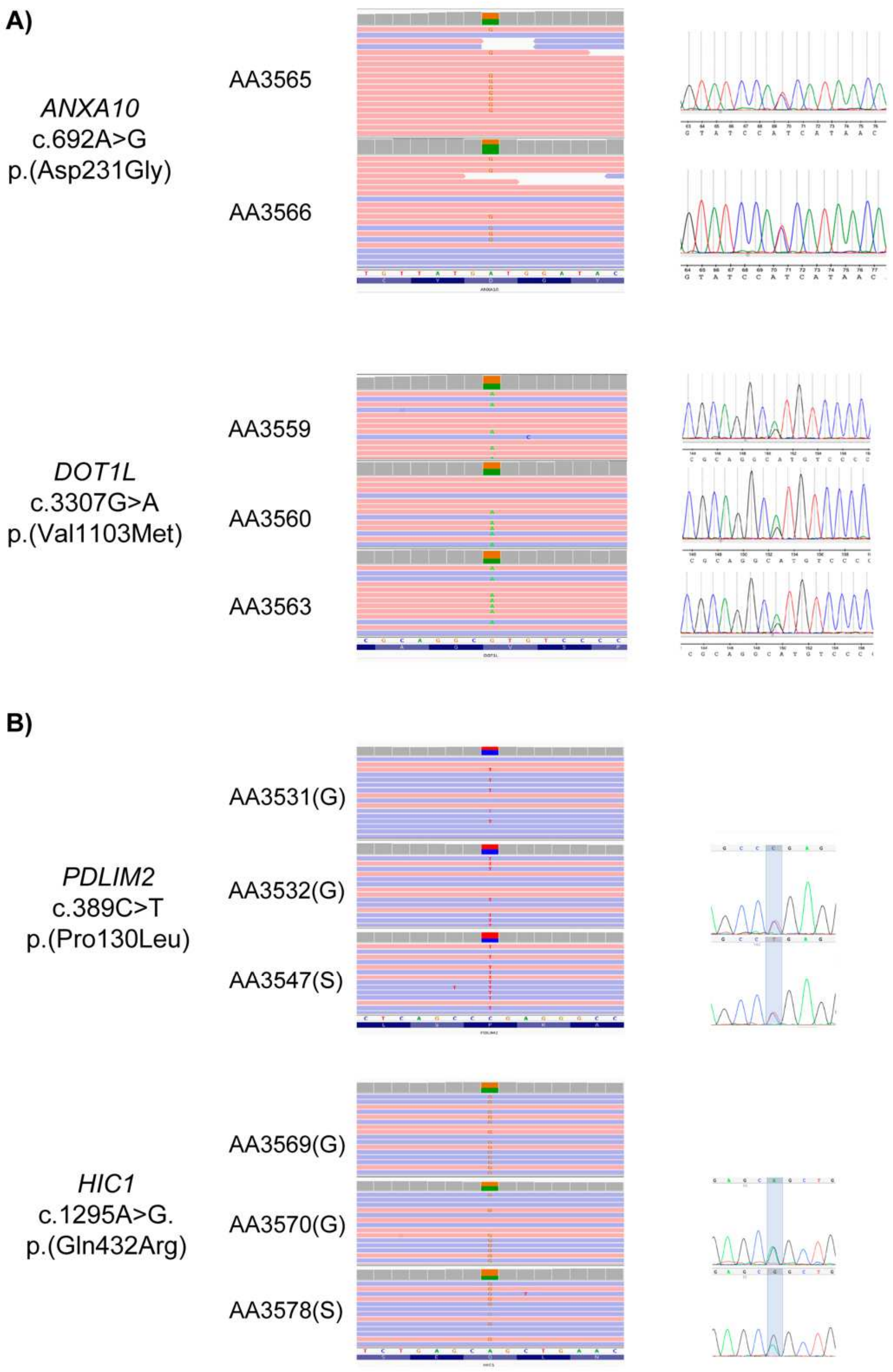

Supplementary Figure 1. A) Sanger sequencing validation of germline variants identified by WES in *ANXA10* (family SPS.12) and *DOT1L* (family SPS.11). Manual inspection using Integrative Genome Viewer (IGV) and the corresponding Sanger sequencing validation. B) Paired germline and somatic WES analysis using ALFRED suggested *PDLIM2* (family SPS.7) and *HIC1* (family SPS.14) as candidate genes undergoing two-hit inactivation. Genetic variants in germline (G) and somatic (S) DNA samples were verified by IGV, and LOH was validated using Sanger sequencing in both germline and somatic samples.
